# A pilot study—genetic diversity and population structure of snow leopards of Gilgit-Baltistan, Pakistan, using molecular techniques

**DOI:** 10.7717/peerj.7672

**Published:** 2019-11-04

**Authors:** Samreen Aruge, Hafsa Batool, Fida M. Khan, Fakhar-i-Abbas, Safia Janjua

**Affiliations:** 1 Institute of Natural and Management Sciences (INAM), Rawalpindi, Pakistan; 2 Centre for Bioresource Research (CBR), Islamabad, Pakistan

**Keywords:** Population, Genetics, *Panthera uncia*, Pakistan, Molecular markers

## Abstract

**Background:**

The Hindu Kush and Karakoram mountain ranges in Pakistan’s northern areas are a natural habitat of the snow leopard (*Panthera uncia* syn. *Uncia uncia*) but the ecological studies on this animal are scarce since it is human shy by nature and lives in difficult mountainous tracts. The pilot study is conducted to exploit the genetic diversity and population structure of the snow leopard in this selected natural habitat of the member of the wildcat family in Pakistan.

**Method:**

About 50 putative scat samples of snow leopard from five localities of Gilgit-Baltistan (Pakistan) along with a control sample of zoo maintained male snow leopard were collected for comparison. Significant quality and quantity of genomic DNA was extracted from scat samples using combined Zhang–phenol–chloroform method and successful amplification of cytochrome c oxidase I gene (190 bp) using mini-barcode primers, seven simple sequence repeats (SSR) markers and Y-linked AMELY gene (200 bp) was done.

**Results:**

Cytochrome c oxidase I gene sequencing suggested that 33/50 (66%) scat samples were of snow leopard. AMELY primer suggested that out of 33 amplified samples, 21 (63.63%) scats were from male and 12 (36.36%) from female leopards. Through successful amplification of DNA of 25 out of 33 (75.75%) scat samples using SSR markers, a total of 68 alleles on seven SSR loci were identified, showing low heterozygosity, while high gene flow between population.

**Discussion:**

The low gene flow rate among the population results in low genetic diversity causing decreased diversification. This affects the adaptability to climatic changes, thus ultimately resulting in decreased population size of the species.

## Introduction

Snow leopard or ounce (*Panthera uncia*) has evolved in the mountainous ranges of Asia (Bonin et al., 2004). Pakistan’s northern mountains are also among its natural habitat, specifically the Gilgit-Baltistan (GB) and Chitral (Khyber Pakhtunkhwa). It is considered an indicator of the health of the ecosystem (O’Brien et al., 1985) and an icon of conservation (Bonin et al., 2004). Though its population in Pakistan has remained constant for the last few years it still needs conservational efforts for its future survival (Sheikh & Molur, 2005). According to recent survey reports, its population may have decreased in the country (Nawaz & Hameed, 2015). Need for studies on its population genetics is emphasized to protect this threatened species (Network, 2014; McCarthy & Chapron, 2003). According to International Union for Conservation of Nature Red List of Threatened Species, it is listed as “Vulnerable” worldwide (McCarthy et al., 2017).

Animals like snow leopards live in difficult field conditions, have small and scattered populations, nocturnal habitat, excellent camouflaging in the wild and human shy nature. These factors contribute toward the difficulties faced by researchers during field sampling for conservation genetic studies (Nowell & Jackson, 1996). Researchers have found a solution by introducing noninvasive sampling which provides a gateway for molecular and genetic analysis, aiming at conservation of wildlife species (Marra et al., 2003; Wolff et al., 2007).

The DNA quantity via scat samples is usually low, but to amplify this low quantity for genetic assessment, simple sequence repeats (SSR) markers are considered excellent, being co-dominant, highly polymorphic and easily transferable between populations (Jarne & Lagoda, 1996; Luo, Hu & Li, 2003). These markers can be easily automated for high throughput screening and information on mutation rate, heterozygosity, homozygosity, number of alleles per locus and Shannon index among different populations (Nei, 1978), while the sex ratio can be determined by using sex chromosome-specific molecular markers. Inbreeding depression, loss of genetic variability and increased homozygosity are associated with smaller populations and reduced chances of species to adapt to the environmental changes (Laurenson & Caro, 1994; Eisen et al., 1998; Keller & Waller, 2002; Chisci, Rossiter & Zappa, 2001; Franklin & Frankham, 1998; Lynch, Conery & Burger, 1995; Slate et al., 2000).

According to a recent estimate, the number of snow leopard individuals is 7,463–7,980 worldwide (McCarthy et al., 2016). Retributive killing, natural prey reduction, human-snow leopard conflicts and illegal trade of bones, pelt and other body parts are the causes of population decline in snow leopards (McCarthy et al., 2017; Maheshwari & Niraj, 2018). Studies conducted on snow leopard populations in central and south–east Asian region including Kyrgyzstan and Tajikistan, India, Nepal, Bhutan, China and Mongolia, show a decreased population size, low genetic diversity and mitochondrial DNA variation making the animals vulnerable to certain environmental conditions (Janečka et al., 2008, 2011, 2017). Keeping in view the conservation status and smaller size of the population of snow leopard, this study aimed at assessing genetic diversity to evaluate the adaptive potentials of the species under environmental changes. The main objective of this study was the optimization of genomic DNA (gDNA) extraction/amplification protocols and use of SSR markers for genetic analysis using field collected scats samples of snow leopard from GB, Pakistan.

## Materials and Methods

### Sample collection

**Figure 1:**
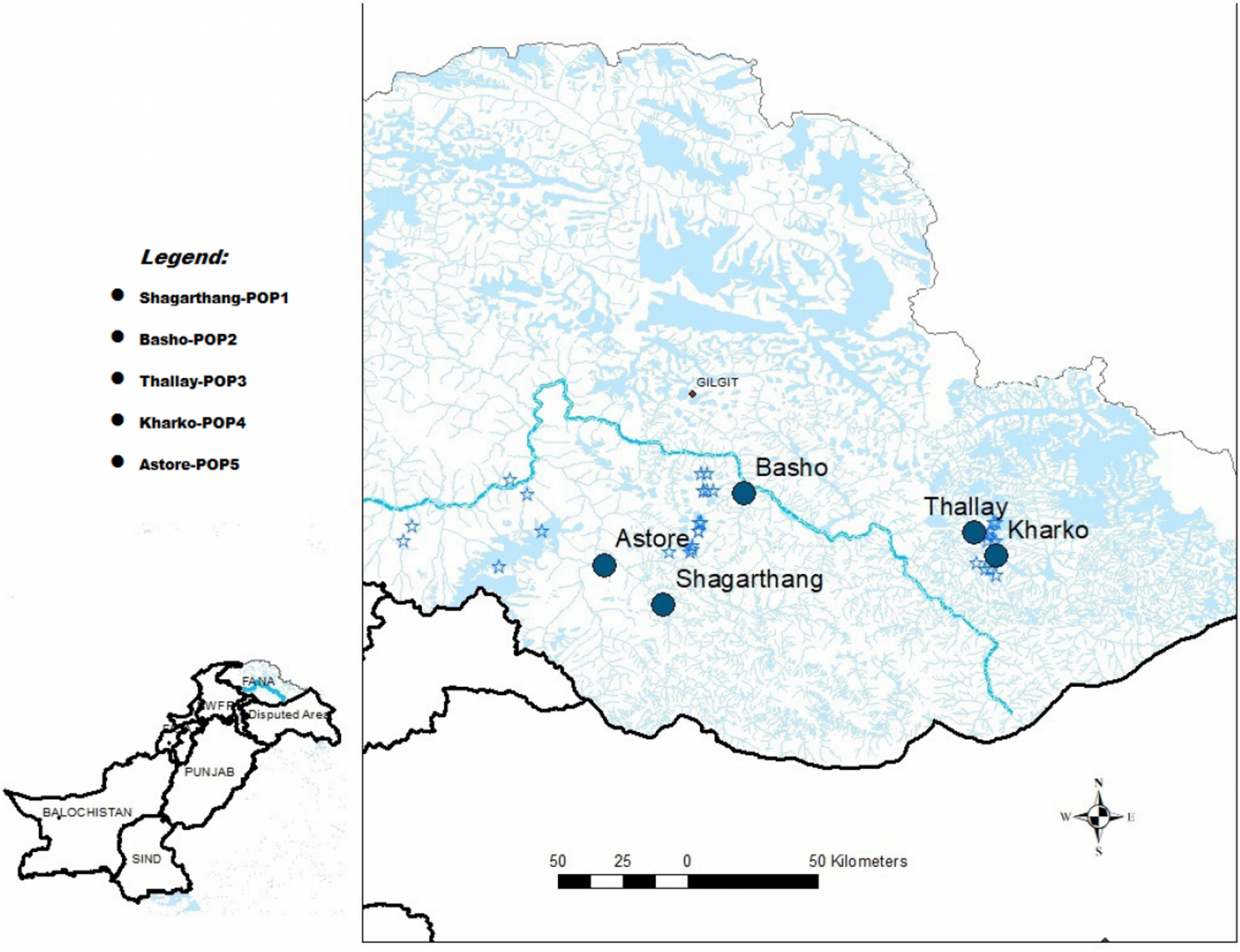
Map of sampling sites. Map of Gilgit-Baltistan showing locations of sampling sites (Map created using ArcGIS Desktop 10.6.1).

Snow leopard scat samples (*n* = 50) were collected between December, 2016 and February, 2017 from five localities of GB (Pakistan), viz., Shagarthang, Basho, Thally, Kharkoo and Astore (Fig. 1). Each sample was packed separately in centrifuge tubes (50 mL) having silica gel to avoid desiccation, labeled and brought to the laboratory and stored at 4 °C till further processing. Samples were subsequently processed for DNA extraction without any delay to obtain good quality of gDNA. A male snow leopard scat sample was provided by Khyber Pakhtunkhwa Wildlife Department (Pakistan), which was used as control.

### DNA extraction

Genomic DNA was extracted from scat samples, using three protocols; (1) Zhang Method: Sample (1.5 g) was washed with ethanol, purified by phenol and gDNA was isolated by binding buffer and column filter (Bernevig, Hughes & Zhang, 2006). (2) Phenol–Chloroform Method: Sample was washed by phosphate buffer saline (PBS), purified by phenol–chloroform and the gDNA was precipitated through ethanol or isopropanol (Wang et al., 2003; Pelaia et al., 2004; Glynn et al., 2004). (3) Combined Zhang and Phenyl-Chloroform Method: Sample (0.25 g) was washed with PBS, bile products were removed by starch and purified with phenol–chloroform, while gDNA was isolated by using binding buffer and filter column. Extracted gDNA was visualized on 1% agarose gel (Sambrook, Fritsch & Maniatis, 1989).

### Amplification

Mini-barcode primers pair having sequence TCCACTAATCACAARGATATTGGTAC and GAAAATCATAATGAAGGCATGAGC (Vaglia et al., 2008) was used to amplify the highly conserved mitochondrial gene, cytochrome c oxidase I (COI), region of extracted gDNA using polymerase chain reaction (PCR). For sex identification, AMELY marker was used (Murphy et al., 1999) after being tested on control male scat sample.

### Microsatellite analysis

Confirmed snow leopard samples were genotyped by using seven microsatellites (SSR) markers, that is, PUN82, PUN100, PUN124, PUN132, PUN225, PUN229 and PUN327 (Janečka et al., 2008). Amplified products were visualized on 2% agarose gel. Bivariate data was generated on the basis of pattern of bands, and preceded through GenAlEx to export data file in PopGen format to calculate allelic frequency, genetic distance, heterozygosity and homozygosity (Nei, 1972).

## Results

### DNA extraction and identification

**Figure 2:**
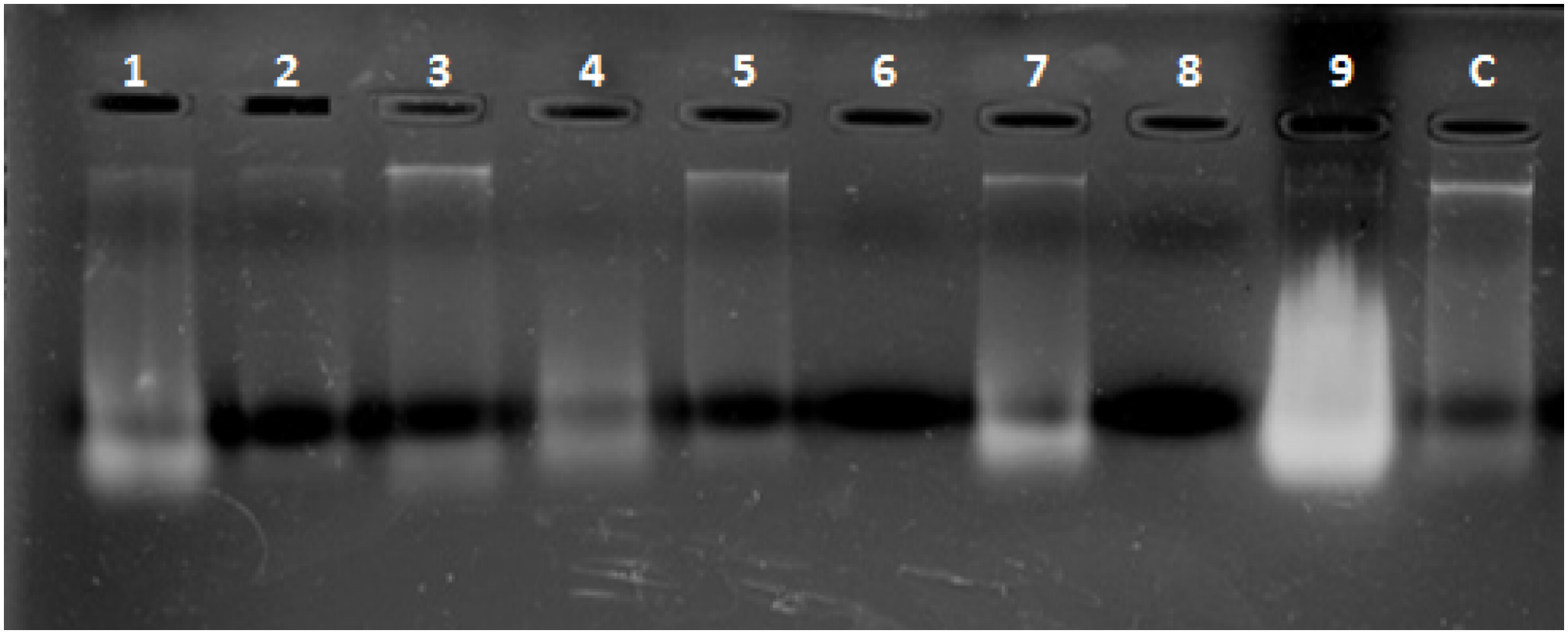
Representative gel plate showing isolated DNA from scat samples. 1–9: scat samples of snow leopard collected from wild, C: control sample from zoo.

**Figure 3:**
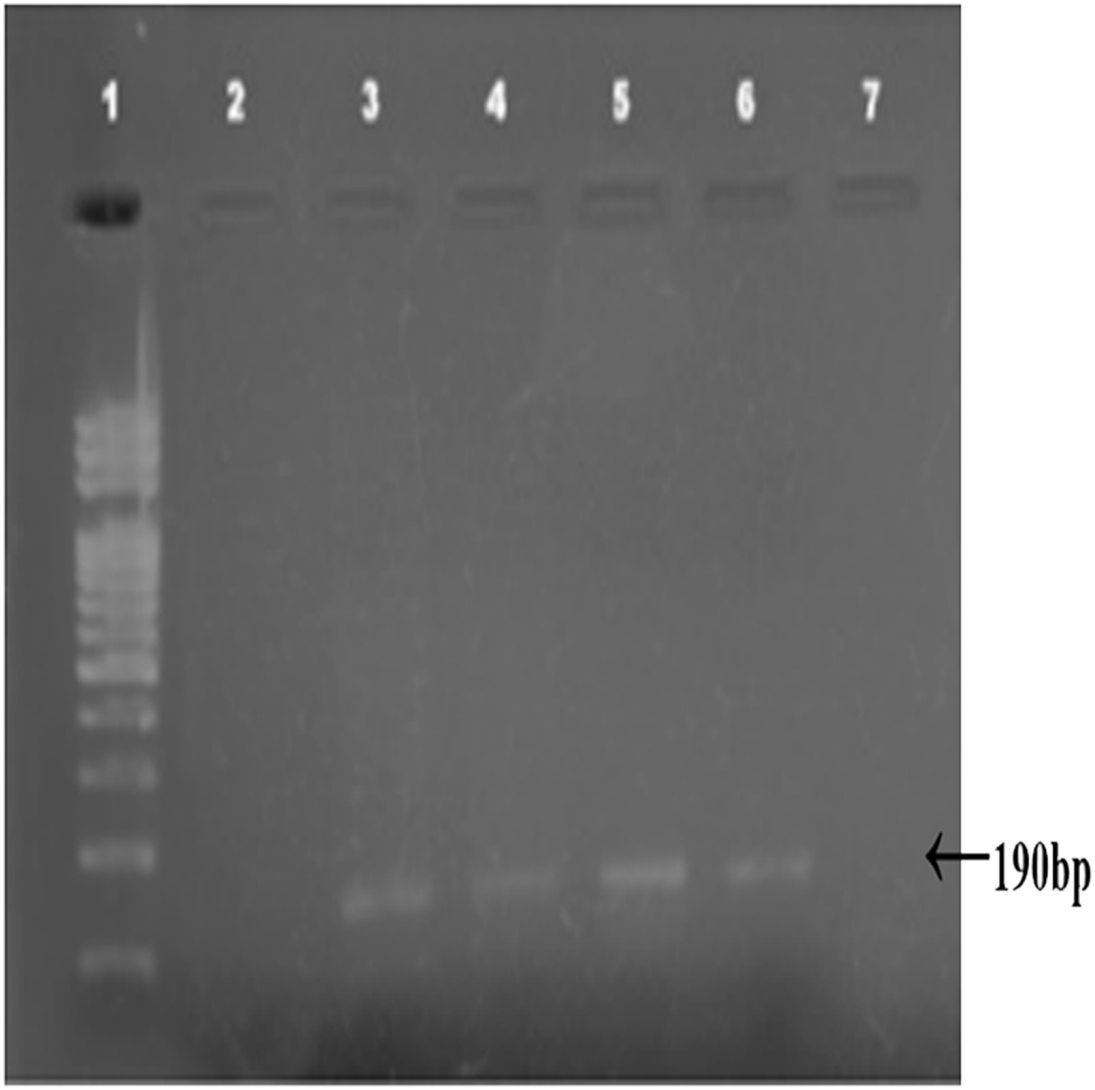
Amplified COI region (190 bp). Amplified COI region (190 bp) by using mini barcode primers; 1: DNA ladder, 2–7: Snow leopard amplified samples.

Combined Zhang–Phenol–Chloroform method was efficient, giving significant extraction success rate, average quantity and quality of gDNA (Table 1; Fig. 2). PCR amplification and sequencing of DNA extracted using 190 bp mini-barcode primer of COI gene (Fig. 3) suggested that 33 out of the total 50 (66%) samples belonged to snow leopard.

**Table 1:** DNA extraction from scat samples of snow leopard.

Methods	Extraction success rate (%)	Average quantity (ng/uL)	Quality (purity ratio)
Zhang	20	1,156	0.7–0.8
Phenol–Chloroform	50	986	<1.2
Combined	67	1,720	1.6–1.8

**Note:**

Success rate, average quantity and quality of extracted DNA from scat samples of snow leopard under different extraction methods.

### Sex determination

**Figure 4:**
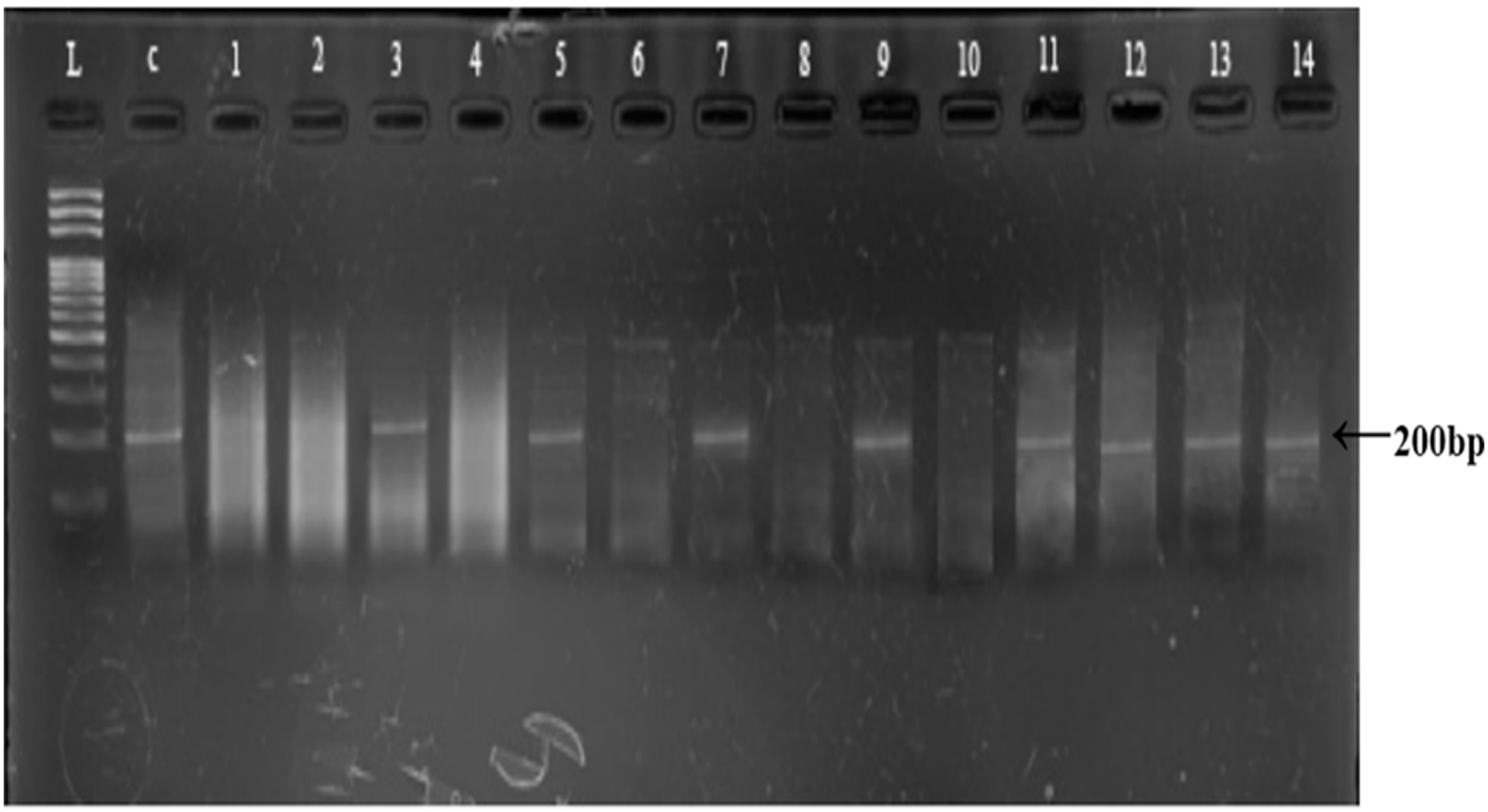
Representative gel of amplified AMELY gene for sex identification. L: 100 bps DNA ladder; C: Control sample, Lane 1–14: amplified/non amplified PCR products.

Polymerase chain reaction amplification of Y-linked AMELY marker (200 bp) was achieved in control male sample and in 21 field collected samples, suggesting a sex ratio of 1.75:1 male to female (21 males and 12 females, Table 2; Fig. 4). The Chi-square test shows that sex ratio in overall samples of snow leopard scats was not significantly different from 1:1 (*p* > 0.05).

**Table 2:** Sex ratio of Snow Leopards. Sex ratio in five populations of Snow Leopard in Gilgit-Baltistan.

Population	Males	Females	Sex ratio (Male:Female)
Shagarthang valley	8	4	2:1
Basho valley	3	2	3:2
Thally valley	3	3	1:1
Kharkoo valley	4	2	2:1
Astore valley	3	1	3:1

### Genetic analysis

The study could amplify 25 samples out of 33 confirmed snow leopard scat samples at annealing temperature of 55 °C for 40 cycles using seven SSR markers. It identified 68 alleles on seven loci with band sizes of 90–220 bp; falling in reasonable proximity with those suggested previously (Janečka et al., 2008). Maximum number (14) of alleles was identified for PUN225 locus and the minimum (6) for PUN82 locus (Table 3).

**Table 3:** Amplified alleles for different SSR markers. Amplified alleles for different SSR markers in comparison with Janečka et al. (2008) study.

Markers	Alleles (#)	Allele size (bp)	
		Present study	Janecka et al.
PUN82	6	100–180	110–115
PUN100	10	90–150	88–96
PUN124	13	90–200	99–100
PUN132	11	90–190	113–123
PUN225	14	100–220	177–183
PUN229	7	90–110	103–113
PUN327	7	90–135	79–91
Overall	68	90–220	79–183

Highest number (44) of alleles were recorded in Shagarthang valley, followed by Thally (33), Astore (27), Basho (26) and the lowest (25) in Kharkoo valley with Shannon indices in different populations ranged between 1.17 and 1.58. Allele frequencies of different SSR loci ranged between 0.02 and 0.75 in different populations (Table 4).

**Table 4:** Allelic frequency and Shannon index among populations. Allelic frequency and Shannon index among five different populations of snow leopard.

Population	Sample size (#)	No. of alleles	Allele frequency	Mean Shannon index (*I*)
Shagarthang	12	44	0.05–0.61	1.58
Basho	5	26	0.10–0.70	1.17
Thally	6	33	0.12–0.62	1.38
Kharkoo	6	25	0.16–0.66	1.17
Astore	4	27	0.12–0.75	1.18

**Figure 5:**
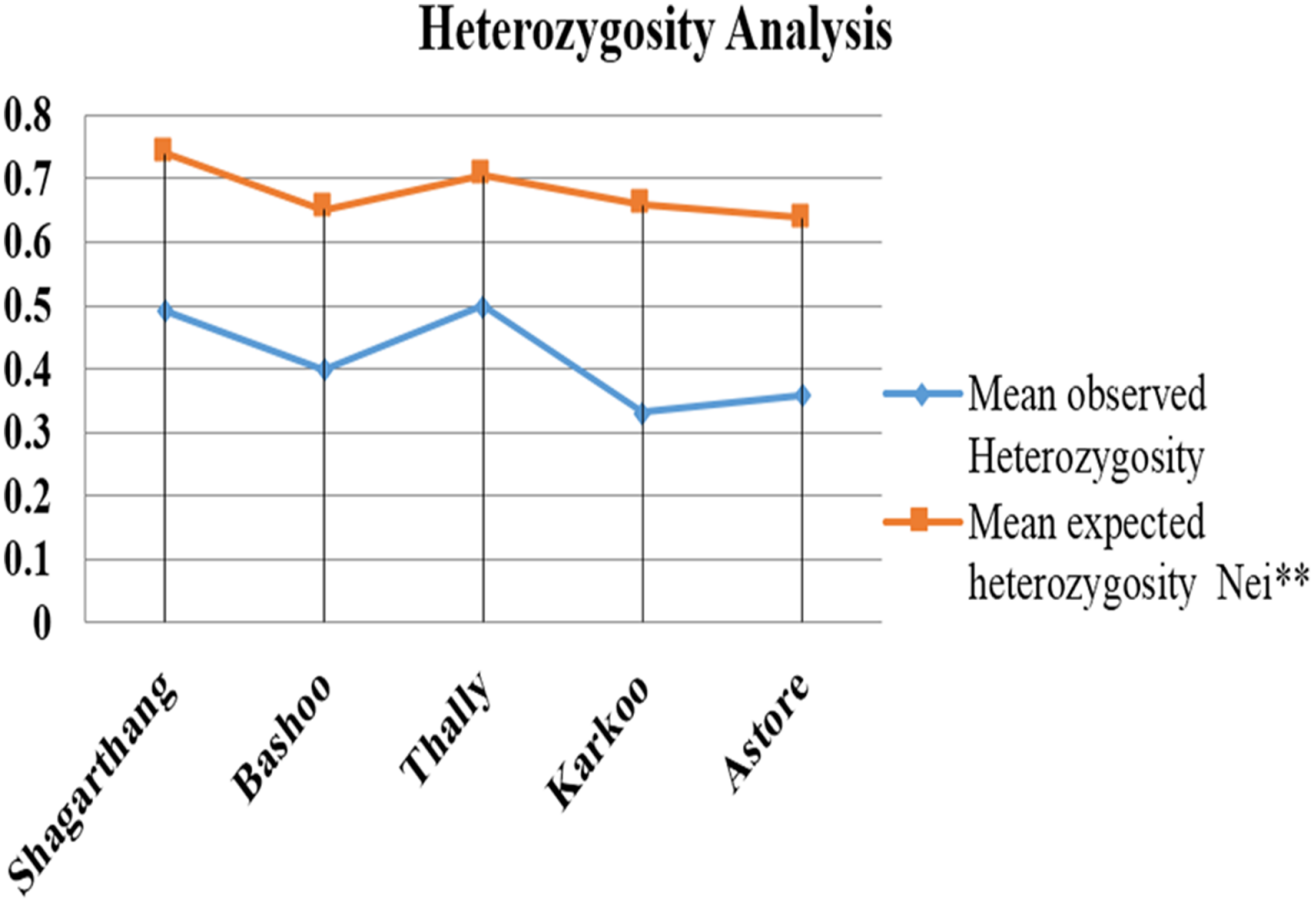
Comparison between mean observed and mean expected heterozygosity in different populations.

Observed heterozygosity (analyzed on PopGen) was lower compared to expected heterozygosity (Fig. 5). Gene flow (Nm), calculated using Wright’s F statistics indices for seven microsatellite primers, reflected high level of gene flow (average 1.65: range 1.18–3.87 for different loci) between populations (Table 5). Nei’s diversity indices of genetic distances (Table 6) between populations were low (<1).

**Table 5:** Fixation indices and gene flow between populations.

Locus	Average deviation in populations (Fis)	Deviation in total population (Fit)	Genetic differentiation (Fst)	Gene flow (Nm)
PUN82	0.26	0.32	0.07	3.21
PUN100	0.54	0.61	0.15	1.40
PUN124	0.64	0.67	0.10	2.21
PUN225	0.45	0.56	0.19	1.05
PUN229	−0.00	0.05	0.06	3.87
PUN327	0.169	0.31	0.17	1.18
PUN132	0.56	0.63	0.15	1.33
Mean	0.38	0.46	0.13	1.65

**Note:**

Fixation indices along with the gene flow between five snow lieoard populations of Gilgit-Baltistan.

**Table 6:** Genetic distances Nei’s diversity indices (above) and genetic distances (below).

Population	Shagarthang	Basho	Thally	Karkoo	Astore
Shagarthang	—	0.67	0.70	0.73	0.70
Basho	0.39	—	0.71	0.56	0.56
Thally	0.34	0.33	—	0.66	0.78
Karkoo	0.30	0.57	0.40	—	0.81
Astore	0.35	0.56	0.24	0.20	—

**Figure 6:**
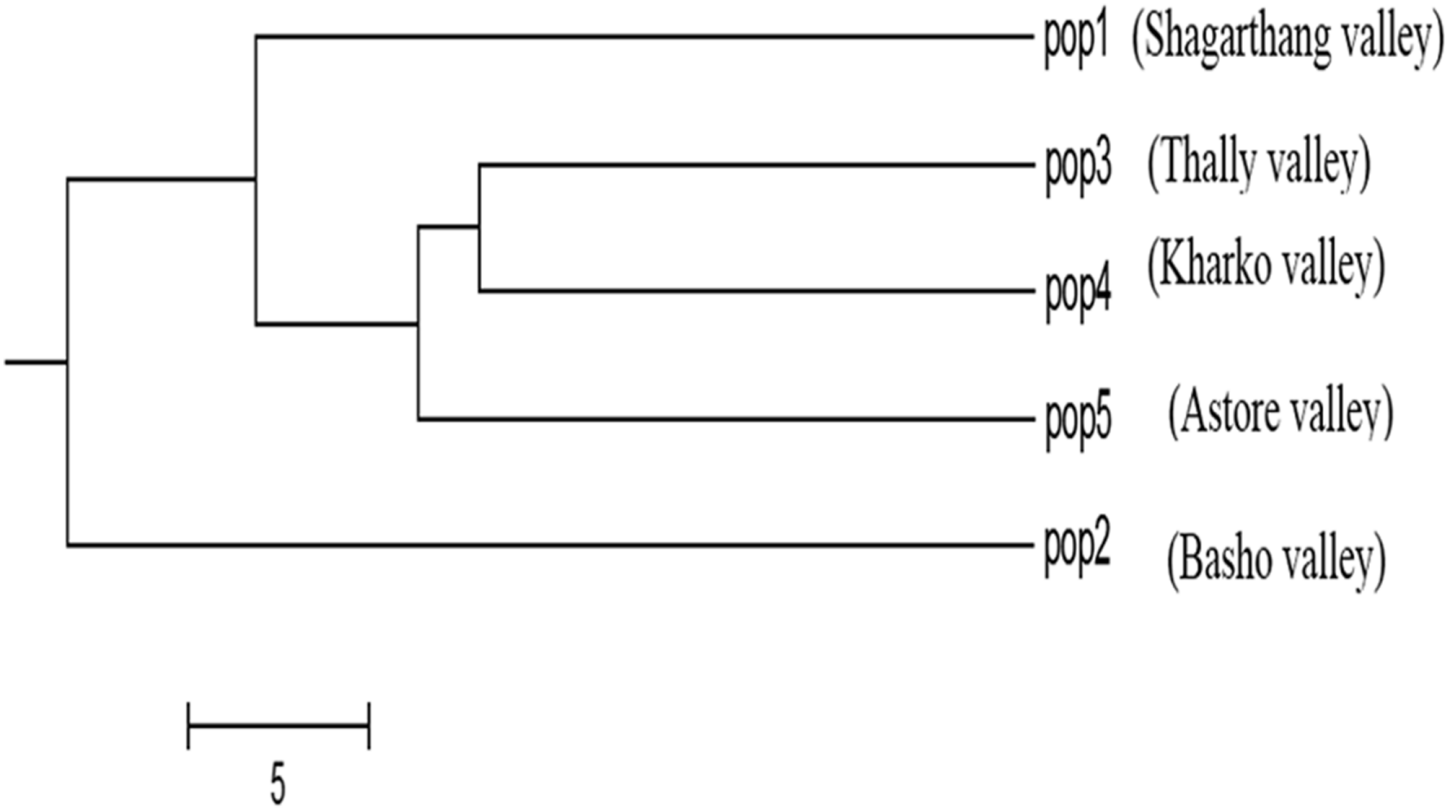
Cluster analysis of five populations of snow leopard based on genetic distances.

**Figure 7:**
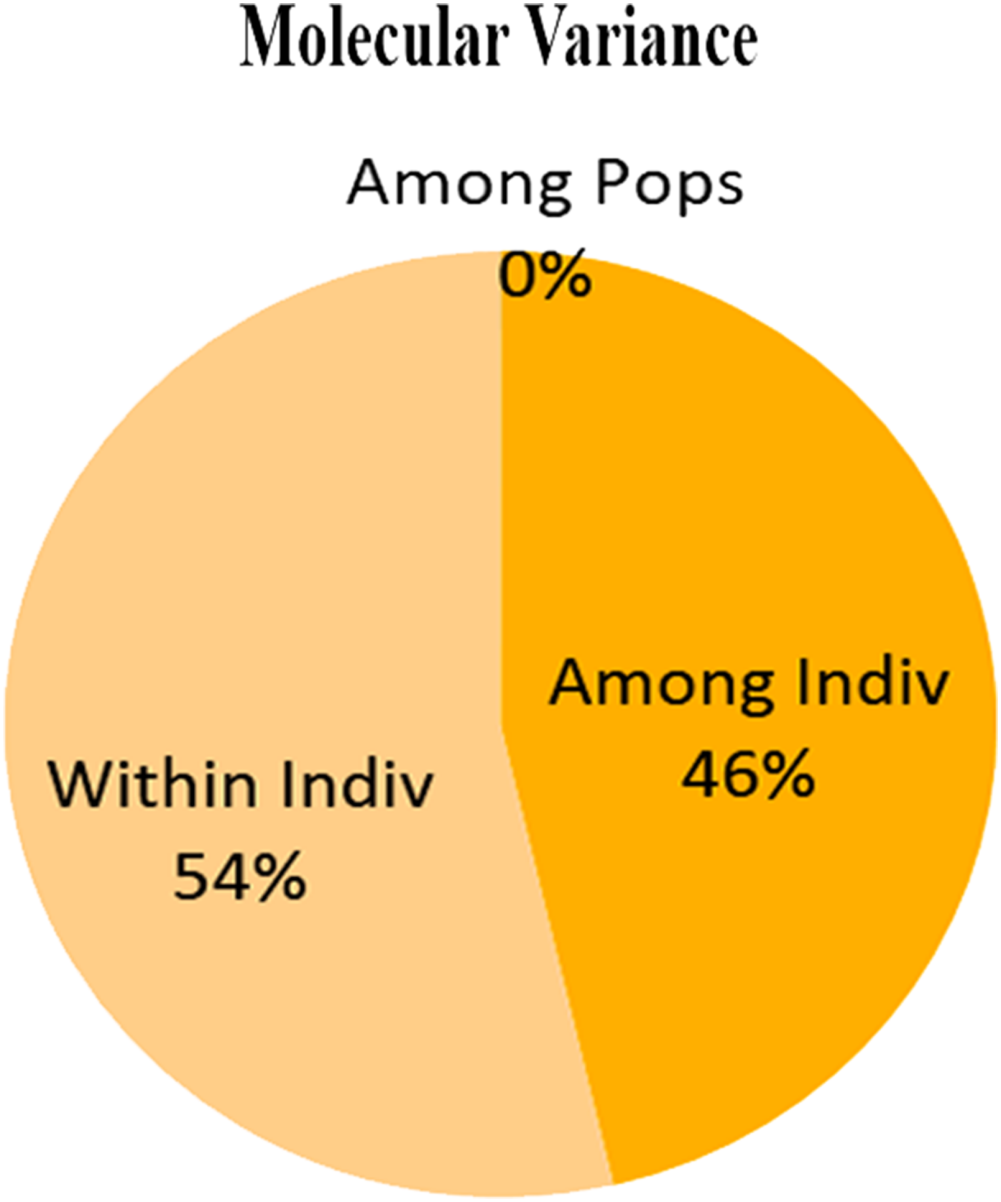
Molecular variance (AMOVA) within and among individuals of snow leopard.

Dendrogram constructed using UPGMA, showed low genetic differences and high genetic similarities among three populations (Thally, Kharkoo and Astore). Shagarthang population exhibited one degree separation from the above three populations, while Basho population appeared as a separate clade (Fig. 6). AMOVA suggested no significant variance among populations indicating no populations’ differentiation among regions. Variance contributed by variability was 46% among individuals and that within individuals was 54%, while 0% was observed among populations (Fig. 7).

## Discussion

In current study DNA was extracted from 50 putative snow leopard scat samples following three protocols (Taberlet, Waits & Luikart, 1999; Frantzen et al., 1998; Murphy et al., 2002; Wasser et al., 1997). Extraction of gDNA from mucosal layer of scat samples using combined Zhang and phenyl-chloroform method (Cobellis et al., 2004; Glynn et al., 2004; Bernevig, Hughes & Zhang, 2006) yielded significant quantity and quality of DNA, which was sufficient for successful amplification of COI gene/mini-barcode, microsatellite and sex determination primers (Thornton et al., 2008; Valledor et al., 2014).

The sample from a control male was amplified by AMELY primer, suggesting the validity of the technique in determination of sex using scat samples of snow leopard. AMELY gene (200 bp) is located on Y-chromosome and hence amplified in males only (Karmacharya et al., 2011; Janečka et al., 2008; Murphy et al., 1999). Out of total 33 confirmed snow leopard scat samples, 21 scats were of male, whereas 12 samples were of female leopards. The possible reason for less number of female leopards could be the ease of their hunting because they usually remain attached with their cubs, so during hunting they cannot escape easily. Also, during post-parturition the vulnerability of female leopards to mortality factors increases because of an increased ecological and energetic cost of parturition and lactation leading to male-biased sex ratio. Gender-biased infanticide could be another cause of less number of females as stated in literature regarding large carnivores (Oli & Rogers, 1996; Whitman et al., 2004; Balme et al., 2013; Bailey, 2005; Balme, Slotow & Hunter, 2009; Swanepoel et al., 2015).

We successfully amplified 75.75% of the field collected scat samples using SSR markers. The lack of amplification in other samples could be due to inhibitors (including bilirubins, bile salts and complex carbohydrates) in the extracted DNA templates (Flekna et al., 2007; Monteiro et al., 2001). The effect of such inhibitors was lowered by using a smaller quantity of the extracted DNA template (Flagstad et al., 2002; Rubin et al., 2000), increasing PCR cycles from 35 to 40 (Frantz et al., 2003; Rubin et al., 2000; Levi et al., 2002; Yekta, hung Shih & Bartel, 2004; Nsubuga et al., 2004; Roon, Waits & Kendall, 2005; Bonin et al., 2004) and using 10× solution “S” as an enhancer (Frackman et al., 1998). Enhancer has the ability to dissolve polar and non-polar compounds resulting in facilitated amplification of DNA (Govinda et al., 2011).

In current study, low genetic variation/heterozygosity (0.33–0.5) was observed in snow leopard population of GB compared to the expected value (0.62–0.75). Low heterozygosity has been reported previously for many populations of snow leopards in Central Asia including Southern Mongolia, Central China, Nepal, north-western India, Pakistan, Tajikistan and Kyrgyzstan (Janjua et al., 2019; Karmacharya et al., 2011; Janečka et al., 2008, 2011, 2017).

The low genetic diversity in these areas caused by smaller isolated size of the population where higher inbreeding results in increased homozygosity and genetic fixation or loss of unexploited genetic potentials (Kotzé & Muller, 1994). It can also be attributed to bottleneck effect, genetic drift and inbreeding depression which sometimes lead to expression of deleterious recessives alleles resulting in lowered survival rate of species (Lindenmayer et al., 1993; Franklin & Frankham, 1998; O’Brien et al., 1985). Small population size resulting in inbreeding depression reduced the chances of adaptation to environmental changes (Barone et al., 1994; Crooks, 2002).

## Conclusion

The findings of genetic diversity, population structure and function played a vital role in formulation of conservational strategies. The low genetic diversity in snow leopard populations of the study leads to the conclusion that the gene flow among the populations is too low and the genetic diversification of the animal is not enough to aptly adapt to the environmental changes which would not result in the efficient promotion of this species. Therefore, proper planning and management in the protected and non-protected areas is required to get the output. The illegal hunting, poaching and human-animal conflicts have to be stopped by arranging proper protective and safety measures to the natural habitat of the inhabitant species in order to maintain the effective population size of the threatened wild fauna.

## Supplemental Information

10.7717/peerj.7672/supp-1 Supplemental Information 1 Allele frequency on different SSR markers analyzed by POP Gen. Allele frequency on different SSR markers at different loci analyzed by POP Gen for snow leopard populations.

## Additional Information and Declarations

### Data Availability

The following information was supplied regarding data availability: Raw data is available in Table S1. The raw data consists of the allele frequency of SSR markers at different loci in snow leopard population samples analyzed by POP Gen.
