# Supplementary material for: A pilot study—genetic diversity and population structure of snow leopards of Gilgit-Baltistan, Pakistan, using molecular techniques
Source: PeerJ. 2019 Nov 4;7:e7672. doi: 10.7717/peerj.7672 (PMC6836756; doi:10.7717/peerj.7672)
Supplement: Supplemental Information 1 — Allele frequency on different SSR markers at different loci analyzed by POP Gen for snow leopard populations. [file peerj-07-7672-s001.docx]

| Allele/Locus | PUN82 | PUN100 | PUN124 | PUN225 | PUN229 | PUN327 | PUN132 |
| --- | --- | --- | --- | --- | --- | --- | --- |
| Allele A | 0.04 | 0.04 | 0.08 | 0.12 | 0.04 | 0.06 | 0.14 |
| Allele B | 0.04 | 0.34 | 0.16 | 0.12 | 0.12 | 0.48 | 0.20 |
| Allele C | 0.20 | 0.16 | 0.02 | 0.16 | 0.24 | 0.22 | 0.02 |
| Allele D | 0.04 | 0.06 | 0.20 | 0.10 | 0.12 | 0.02 | 0.22 |
| Allele E | 0.14 | 0.10 | 0.02 | 0.04 | 0.42 | 0.02 | 0.08 |
| Allele F | 0.06 | 0.02 | 0.06 | 0.12 | 0.06 | 0.16 | 0.02 |
| Allele G | 0.46 | 0.24 | 0.02 | 0.04 | …….. | 0.04 | 0.22 |
| Allele H | 0.02 | 0.02 | 0.14 | 0.06 | …….. | ……….. | 0.02 |
| Allele I | ……….. | 0.02 | 0.04 | 0.08 | …….. | ……….. | 0.02 |
| Allele J | …………. | …………. | 0.22 | 0.14 | ……... | ……….. | 0.06 |
| Allele K | …………. | ……….. | 0.02 | 0.02 | ……... | ………… | ……….. |
| Allele L | ………… | ………… | 0.02 | …….. | ……... | ………… | ……….. |
